# When practice outpaces policy: Whatsapp use among nursing and medical staff in Israeli hospitals

**DOI:** 10.1186/s13584-026-00754-3

**Published:** 2026-04-02

**Authors:** Drora Ben Michael Vinker, Maya Peled-Raz

**Affiliations:** 1 School of Public Health, Faculty of Social Welfare and Health Sciences, University of Haifa, Haifa, Israel; 2 Hillel Yaffe Medical Center, Hadera, Israel

**Keywords:** WhatsApp, Data security, Medical confidentiality, Health policy, Team communication, Regulation, Medical technology, Digital communication.

## Abstract

**Background and objectives:**

WhatsApp has become a central communication tool among medical and nursing staff worldwide, offering a fast and convenient way to share clinical information. However, its widespread use raises concerns regarding patient confidentiality and data security. This study examined the extent and characteristics of WhatsApp use for clinical purposes among hospital-based healthcare professionals in Israel, and the perceived risks and benefits associated with its use.

**Methods:**

A quantitative cross-sectional study was conducted using a structured questionnaire adapted from De Benedictis et al., among 283 participants – 132 medical staff and 151 nursing staff – from hospitals across Israel. The questionnaire assessed usage patterns, perceived utility, and perceived risk. Data were analyzed using hierarchical regression to identify predictors of use.

**Results:**

Professional WhatsApp use was nearly universal (reported by 100% of physicians and 97.4% of nurses). Physicians reported significantly higher use in both non-identifiable data sharing (mean 3.46 on a 1–5 scale, with 1 = strongly disagree, 5 = strongly agree) and identifiable data sharing (mean 2.61) compared to nurses (means 2.96 and 2.26, respectively). Perceived personal benefit was high (mean 5.19 on a 1–7 scale, with 1 = strongly disagree, 7 = strongly agree), while perceived organizational support was low (mean 1.86), indicating a lack of official guidelines. Regression analysis identified four predictors: age (negative association), and personal, normative-organizational, and regulatory-organizational benefits (positive associations). Perceived risk was higher among women and negatively predicted use.

**Conclusions:**

WhatsApp is a deeply embedded communication tool in the daily clinical practice of the surveyed medical and nursing staff. This reflects a “normalization of non-compliance”, where the perceived efficiency of WhatsApp overrides regulatory prohibitions. Given its advantages and the current policy ambiguity, there is an urgent need for balanced regulatory guidance that leverages built-in security features, enhances risk awareness, and aligns with real-world practices to ensure both efficiency and patient privacy.

**Public interest summary:**

WhatsApp is widely used by healthcare professionals to share clinical information quickly and efficiently. Our study found that almost all surveyed physicians and nurses use the app for work-related communication, and that identifiable patient information is often shared, despite formal policies prohibiting it. While hospitals issue guidelines that discourage such use, these have limited impact. In contrast, strong social and professional norms within teams encourage WhatsApp communication, making it an integral part of hospital life. The findings mirror global research showing that the need for fast, accessible communication often overrides institutional restrictions, reflecting a “normalization of non-compliance”. This pattern highlights a universal policy gap: WhatsApp and similar apps have become essential tools in healthcare systems worldwide yet are used without adequate regulation. The study calls for practical, balanced policies that align with real-world clinical needs while ensuring the protection of patient privacy.

**Supplementary Information:**

The online version contains supplementary material available at 10.1186/s13584-026-00754-3.

## Introduction

In the past decade, technological developments have led to a revolutionary change in the nature of communication within the healthcare system. The smartphone has become a central tool for communication among members of medical teams, primarily through instant messaging applications such as WhatsApp, which allow for accessible, simple, and cost-free communication [1]. WhatsApp, founded in 2009, has become the most popular free communication application of the 21 st century [2]. In 2020, it reached two billion users worldwide across more than 180 countries [1, 3]. The number of WhatsApp users exceeds the number of desktop computer users [4]. The use of WhatsApp has also become very common in the workplace [4]. The application enables the transfer of medical information in a fast, convenient, and efficient manner, thereby contributing to the acceleration of decision-making processes and the improvement of the quality of care [5] allowing information sharing and earlier initiation of medical treatment [6].

WhatsApp promises a simple, secure, and reliable messaging service. Since 2016, the application has offered end-to-end encryption, ensuring that only the communicating users can read the messages – not any third party, not even WhatsApp itself [2]. However, this encryption does not address the security of data stored at the endpoints – that is, data saved on users’ smartphones or in their cloud storage.

The use of instant messaging applications in the healthcare system is highly prevalent. A study conducted among medical teams in a UK teaching hospital found that WhatsApp is the primary application used by staff [7]. A comprehensive literature review showed that WhatsApp is the most popular application among clinicians and was mentioned in 42% of the articles reviewed [8]. These results are consistent with other reports indicating that WhatsApp usage is perceived as improving and streamlining case management through better and faster decision-making [2, 9].

## Communication Challenges in the Healthcare System

Professional communication between and within teams is critical in medical care and must be open, collaborative, and responsible in order to provide the best care for patients [2]. In hospitals, communication challenges include multidisciplinary teams, complex hierarchies, an abundance of clinical information, and the critical importance of response time [10]. Teams that create WhatsApp groups promote communication and collaboration, which in turn are important for enhancing the level of healthcare services provided to patients [5].

In the past, communication between physicians took place through face-to-face meetings, telephone, pager, or email, which are cumbersome and time-consuming tools [2]. The pager, which was the classic form of communication in hospitals, is indeed reliable and inexpensive, but it leads to time wastage due to the need for follow-up phone calls [11]. In contrast, communication via WhatsApp is easy, accessible and enables the transfer of clinical information – including visual information (photos and videos) – in a fast and direct manner between caregivers [12].

The literature indicates that nursing staff use smartphones during work to communicate with team members through text messages and voice messages [13]. Likewise, a study conducted among internal medicine residents in the United States found that the residents preferred WhatsApp communication within the hospital over communication via telephone, email, or pager. Approximately 80% of the residents responded that WhatsApp communication was their preferred method when considering efficiency and ease of use [14]. In addition, it was found that intuitive interfaces on the smartphone device and the user’s ability to successfully operate the device, control it, and receive clear alerts and information from the device help enhance users’ sense of security [15].

Alongside the many advantages, questions arise regarding the preservation of medical confidentiality, information security, and the real concern over the exposure of sensitive personal information [5]. In a study among medical teams in hospitals in Australia, 67% of respondents perceived the application as only “moderately secure”, and only 21% believed that the information on it was secure [10]. Evidence from EU countries indicates that sensitive patient data is frequently transmitted over inadequately secured digital platforms, with healthcare providers often falling short of the information security standards mandated by the General Data Protection Regulation (GDPR) [16, 17]. Similarly, studies have shown that future healthcare professionals demonstrate low perceived susceptibility to mobile security breaches, despite recognizing their potential severity [18].

### WhatsApp Usage and Regulatory Challenges in the Israeli Healthcare Ecosystem

Israel presents a unique case study regarding the adoption of instant messaging. Recent data indicates that WhatsApp is the most dominant social platform in the country [19]. In the healthcare sector, this trend is even more pronounced; a study based on data from 2019 to 2020 by Barayev et al. found that over 86% of primary care physicians and specialists use the application daily for professional consultations [20]. While explicitly acknowledging the potential for confidentiality breaches, physicians continue to rely on the platform due to its efficiency and availability.

Protection of privacy is a fundamental value In the State of Israel, enshrined in the “Basic Law: Human Dignity and Freedom” [21] as well as the “Privacy Protection Law” of 1981 [22]. In addition, Sect. 19 of the “Patient’s Rights Law” specifically stipulates a duty of medical confidentiality that applies to every caregiver or employee in a medical institution [23]. Despite the importance of the issue, until recently there were no guidelines or clear regulatory references regarding the use of instant messaging applications in the Israeli healthcare system.

In January 2020, the Ministry of Health published an ethical code for maintaining medical confidentiality and protecting personal information privacy in the healthcare system. According to the ethical code, personal information should not be disseminated in the media or social media even if it is not identified in a way that could lead to identifying the person [24]. The publication did not include concrete implementation guidelines.

The first reference directly relevant to our issue of appears In Edition 7 of the JCI (MOI.12 standard), published in June 2021, requiring Israeli hospitals to formulate institutional policy ensuring the security and documentation of information transmitted through instant messaging applications [25].

The relevant Israeli regulatory landscape began to evolve significantly in 2022, addressing the issue from two complementary angles: organizational infrastructure and patient privacy. First, the Ministry of Health issued **Circular 06/2022**, the ‘Cyber Defense Regulation’ [26]. This comprehensive directive focuses on the **institutional level**, requiring healthcare organizations to appoint cyber defense officers and implement advanced security strategies to protect critical infrastructure and central databases. While crucial for preventing large-scale cyberattacks, this regulation focuses on managed organizational systems and does not provide specific protocols for the daily use of personal smartphones by staff members.

Concurrently, the Privacy Protection Authority at the Ministry of Justice published guidelines regarding **Telemedicine Services (Amendment 13)** [27]. This document addressed the privacy risks arising from remote medical consultations (video/audio). It established a clear principle: medical information should not be stored on private devices. The guidelines instructed that if a personal device is used for a consultation, the information must be deleted immediately upon completion. However, its primary focus was on formal telemedicine sessions rather than the informal, asynchronous text messaging that dominates daily hospital work.

It was only in 2024 that a direct regulatory reference to instant messaging applications was published. In this specific document, the Privacy Protection Authority addressed the “Transfer of Medical Information via Digital Means and Non-Dedicated Software” [28]. This guideline explicitly identifies the use of commercial apps (such as WhatsApp) on personal devices as a severe security risk. It determines that such usage should be avoided whenever possible. When no dedicated alternative exists, it imposes strict operational limitations: strict de-identification of data (removing name, ID, etc.) and immediate deletion of the content from the personal device after it is documented in the medical record.

Accordingly, while healthcare organizations are now formally required to act with a “proactive approach” [26], the practical reality for the field clinician remains problematic. The current regulations primarily emphasize prohibitions (what *not* to do) and risk warnings, rather than offering a functional, user-friendly, and secure alternative that can match the efficiency of WhatsApp. Therefore, a significant gap remains between the formal policy and the actual practices in the field.

Therefore, the current study seeks to examine the extent and nature of WhatsApp use among physicians and, for the first time, nurses working in hospitals in Israel, and is the first of its kind to investigate the perceptions of Israeli medical and nursing staff regarding the risks and advantages associated with its use.

## Methods

### Theoretical Framework

A quantitative research design was employed based on a validated questionnaire developed by De Benedictis et al. [29], and adapted for the purposes of the current research. The questionnaire is grounded is two theoretical models. The first is the Technology Acceptance Model (TAM), which examines the factors prompting individuals to accept or reject information technologies [30]. The TAM model identifies two primary explanatory variables for technology adoption: perceived usefulness and perceived ease of use. Perceived usefulness refers to the extent to which a technology is believed to improve performance and facilitate better outcomes, while perceived ease of use reflects the perceived effort (in terms of time, cost, and exertion) required to operate the technology [31].

The Second is the Institutional Theory, which explains how organizational forces shape the behavior of institutions and professionals. Scott’s Institutional Theory outlines three “institutional pillars” that influence organizational behavior: The **regulatory dimension** – the presence of regulations, laws, and monitoring mechanisms within the organization, where violations lead to sanctions; The **normative dimension** – the social norms of appropriate behavior and the desire of individuals to align with what is considered “proper” conduct; and The **cultural-cognitive dimension** – the shared understandings and cultural assumptions embedded in the organization which are taken for granted [32].

The integrated theoretical model was developed to assess the interplay between individual and organizational variables. Based on the study’s research questions, the framework assumed that both personal and institutional factors contribute to explaining WhatsApp use among healthcare professionals in hospital settings.

### Study population

The study population comprised medical and nursing staff employed in hospitals across Israel. Data collection occurred between June and November 2023, using a multi-stage hybrid sampling strategy. The first stage utilized convenience sampling through the distribution of a digital survey link in professional WhatsApp groups (including both local hospital-specific groups and national professional forums). To reach a broader population, respondents were encouraged to forward the survey link to colleagues, incorporating a snowball sampling method. In the second stage, to mitigate selection bias and reach personnel who may be less responsive to digital platforms, a supplementary convenience sample was collected using printed questionnaires distributed in specific wards (e.g., internal medicine and surgery) of a tertiary medical center. To ensure anonymity for those using the printed format, respondents were instructed to deposit completed forms into sealed collection boxes. Duplicate responses were prevented by restricting digital submissions to one per user and verifying non-participation prior to manual distribution.

Based on prior research indicating a WhatsApp usage rate of at least 70% [2], the required sample size was calculated using G*Power software with a significance level of α = 0.05 and statistical power of 0.80 [10, 29, 33, 34]. The calculated sample size was 274 participants (137 medical staff and 137 nursing staff). In practice, 283 participants were included in the final analysis after excluding two incomplete responses – 132 from medical staff and 151 from nursing staff. Of the questionnaires, 29% (*n* = 82) were completed manually (printed forms).

#### Research Tool

The research tool was based on validated scales from the questionnaire developed by De Benedictis et al. [29], which were adapted for the current study and the Israeli context. Specifically, the instrument integrates validated measures for the core theoretical variables (Perceived Usefulness, Perceived Risk) with context-specific measures (Regulatory and Normative factors) that were modified to fit the local legal and cultural environment.

The adaptation process included several steps. First, a preliminary interview was conducted with a hospital information security officer to ensure regulatory alignment. Next, content validity was evaluated by three experts. To ensure linguistic and conceptual equivalence, an independent back-translation procedure was employed. Finally, a pre-test pilot was conducted among 5 healthcare professionals (2 physicians and 3 nurses), which included interviews to verify item clarity and relevance. Based on the feedback, minor refinements were made to the questionnaire.

The questionnaire comprised 59 items organized into five sections (retaining the original Likert scaling of the adapted instrument): (A) Demographic questions; (B) Extent and nature of WhatsApp use for personal and professional purposes (Likert scale 1–5, with 1 = strongly disagree, 5 = strongly agree); (C) Perceived professional utility (Likert scale 1–7, with 1 = strongly disagree, 7 = strongly agree); (D) Perceived organizational utility – regulatory factors (Likert scale 1–5) and normative factors (Likert scale 1–7); (E) Perceived risk (Likert scale 1–7).

Participants were also asked about the types of information transmitted via WhatsApp, the security measures used on their smartphones, and their familiarity with relevant guidelines and procedures. These questions were informed by emerging regulatory discussions in Israel and reviewed by clinical and legal experts to ensure content validity.

The dependent variable, WhatsApp use, was assessed using items 10–24 and demonstrated high internal reliability (Cronbach’s α = 0.895). The independent variables also showed high internal consistency: Perceived personal professional utility: items 25–35 (α = 0.875); Perceived organizational utility – regulatory factors: items 36–39 (α = 0.679); Perceived organizational utility – normative factors: items 40–44 (α = 0.743); Perceived risk: items 45–59 (α = 0.886). It should be noted that while the alpha value for the locally adapted regulatory factors (0.679) is slightly below the conventional 0.70 threshold, it is considered acceptable for exploratory research in social sciences, particularly given the small number of items in this sub-scale.

The questionnaire was administered in Hebrew. An English translation is provided as *appendix 1.*

##### Statistical Analysis

Digital demographic fields were mandatory. Two incomplete questionnaires were excluded, and sporadic missing values were coded as ‘missing’ for statistical analysis. Statistical analysis was conducted using IBM SPSS software version 28. Descriptive statistics were calculated, including frequencies, means, and standard deviations. Associations between variables were examined using t-tests and Pearson correlations. One-way analysis of variance (ANOVA) was performed to evaluate differences across data sources.

To predict WhatsApp use, a two-step hierarchical linear regression was conducted. The dependent variable was calculated as the mean score of the 15 items assessing professional usage (items 10–24), treating it as a continuous variable ranging from 1 to 5. Prior to the regression analysis, multicollinearity among independent variables was tested, and assumptions of normality and homoscedasticity were assessed.

## Ethical Considerations

The study received approval from the Ethics Committee of the University of Haifa (Approval No. 007/23). Participation was entirely voluntary, and the questionnaire was anonymous, collecting no identifying information. Informed consent was obtained through a detailed explanatory statement that preceded the questionnaire. This statement outlined the purpose of the study, the voluntary nature of participation, and participants’ rights, including the right to withdraw at any time. Only after reading this information did participants choose whether to proceed with completing and submitting the questionnaire, a process which was considered to constitute informed consent.

## Results

### Sample characteristics

The study included 283 healthcare professionals, of whom 132 (46.6%) were physicians and 151 (53.4%) were nursing staff. Of participants, 62.5% were women, with a notable difference between professional groups: 75.7% of the nursing staff were women compared to 47.4% of the medical staff. This ratio closely reflects the actual gender distribution in the healthcare workforce, where women comprise approximately 85% of nursing staff and 43% of physicians [35]. Age differences were also observed: the medical staff were relatively younger, with 59.4% under the age of 40, compared to only 38.2% among the nursing staff. Most participants (86.7%) worked in general government hospitals, while the remainder were employed in non-governmental general hospitals. The distribution of participants by job roles was balanced across groups: among medical staff – managerial positions, specialists, residents, and interns; among nursing staff – managerial positions, team leaders, and regular staff, barring headquarters staff, who represented only 8.6% of the sample – consistent with their representation in the general target population.

Full demographic characteristics of the sample are presented in Table 1.

**Table 1:** Socio-demographic Background Characteristics (*N* = 283)

Variable	Total (*N* = 283)		Medical Staff (*N* = 132)		Nursing Staff (*N* = 151)	
	*N*	%	*N*	%	*N*	%
**Gender**						
Male	105	37.5	69	52.6	36	24.3
Female	178	62.5	63	47.4	115	75.7
**Profession**						
Physician	283		132	46.7		
Nurse					151	53.3
**Age Groups**						
21–30	38	14	26	20.3	12	8.6
31–40	97	34	52	39.1	45	29.6
41–50	64	22.5	21	15.8	43	28.3
> 51	84	29.5	33	24.8	51	33.6
**Years of Experience**						
0–5	82	29.5	59	45.2	23	15.8
6–10	46	16.1	24	18	22	14.5
11–15	38	13.3	12	9	26	17.1
> 16	117	41.1	37	27.8	80	52.6
**Position**						
Managerial position			36	27.3		
Specialist			30	22.7		
Resident			43	32.6		
Intern			23	17.4		
Headquarters staff					39	8.6
Managerial position					41	25.8
Nursing team leader					58	27.2
Nursing staff					13	38.4
**Medical Divisions**						
Surgical + OR	120	42.8	41	31.6	79	52.7
Internal + ER+Pediatric	95	33.4	65	48.9	30	19.7
Maternity and Obstetrics	30	10.5	5	3.8	25	16.4
Other	38	13.3	21	15.8	17	11.2

### Whatsapp usage patterns

WhatsApp use was found to be extremely prevalent among participants, with 98.6% of participants reporting high frequency of personal use (Mean = 4.93, on a 1–5 Likert scale,, with 1 = strongly disagree, 5 = strongly agree, SD = 0.35). Similarly, nearly all respondents (98.59%) reported using the app to exchange clinical information, with 100% of physicians and 97.4% of nurses reporting professional use. The types of information most commonly shared included: posing clinical questions to colleagues (79.4%), providing professional responses (77.6%), sharing work-related images (71.9%), discussing administrative matters (65.6%), transferring patient identification stickers (51%), and sharing laboratory test results (48.2%).

Although use of WhatsApp was common across both groups, physicians reported significantly higher usage across all categories. For instance, 85.6% of physicians reported sharing work-related images compared to only 59.6% of nurses. Similarly, 84.8% of physicians said they posed questions to colleagues via WhatsApp, compared to 74.2% of nursing staff.

Overall professional use of WhatsApp was rated at a moderate level (mean score of 2.9 on a 1–5 Likert scale, with 1 = strongly disagree, 5 = strongly agree), with some variability depending on the type of use. The most common use in both groups was for sharing scientific information with peers (mean 3.61 for nurses and 3.68 for physicians).

A statistically significant difference (α = 0.000) was observed in the use of WhatsApp for “discussion of clinical cases without identifying information”: 86.4% of physicians reported such use, compared to 64.3% of nurses. Another significant difference (α = 0.024) was found in the transmission of identifiable patient data: 41.1% of nurses reported they never shared identifiable information, compared to only 22.7% of physicians.

Detailed usage patterns are presented in *Table S1 in Appendix 2.*

### Perceived professional utility

Professional utility was assessed using 11 items, with descriptive statistics reported in *Table S2 in Appendix 2.* Participants reported high perceived utility in three areas: time saving (mean 5.46 for nurses, 5.89 for physicians); improved communication among caregivers (mean 5.27 for nurses, 5.73 for physicians); and preference over other apps due to broad user adoption (mean 5.21 for nurses, 5.48 for physicians).

Comparative analysis revealed significant differences between professional groups. Physicians reported significantly higher perceived utility than nurses in terms of time savings (α = 0.012) and improved communication (α = 0.012). Physicians also expressed greater willingness to use personal smartphones for work purposes (α = 0.014).

### Perceived organizational regulatory utility

Organizational utility, stemming from regulatory factors, was measured using four items (see *Table S3 in Appendix 2*). Overall, perceived regulatory support for WhatsApp use was very low (mean 1.86 on a 1–7 scale, with 1 = strongly disagree, 7 = strongly agree). However, notable differences were found between groups: a higher proportion of physicians (79.5%) than nurses (57%) reported that hospital management had never prohibited WhatsApp use among colleagues (α = 0.000). About one-third of participants indicated they had never encountered an explicit managerial ban on transmitting sensitive patient data.

Nonetheless, 71.3% of nurses reported the issue of such a ban, compared to only 61.3% of physicians (α = 0.010), indicating that many continue to use WhatsApp despite awareness of institutional opposition. Conversely, 49.7% of nurses reported feeling organizational pressure to use WhatsApp, compared to 34.1% of physicians (α = 0.001). These findings suggest that nurses are more influenced by management’s position – in terms of both restrictions and implicit coercion.

### Perceived organizational normative utility

Normative utility was measured using items 40–44, with descriptive statistics in *Table S4 of Appendix 2.* Perceived utility from normative influences was high across both groups (mean 5.5 on a 1–7 scale, with 1 = strongly disagree, 7 = strongly agree), suggesting strong informal norms supporting WhatsApp use.

Nearly all participants (95%) believed that their colleagues use WhatsApp for personal purposes, indicating that the app is deeply embedded in daily life. However, significant group differences were found in perceptions of professional use: 90.9% of physicians versus 82% of nurses believed their peers used WhatsApp for professional reasons (α = 0.005). Similar gaps were found in perceptions of scientific information sharing (87.2% of physicians vs. 76.7% of nurses, α = 0.004) and especially the sharing of identifiable clinical data (75.7% of physicians vs. 56.6% of nurses, α = 0.000).

These results suggest diverging professional norms between doctors and nurses regarding the appropriateness of using WhatsApp in clinical settings.

### Perceived risk

Perceived risk was measured using items 45–59 (see *Table S5 in Appendix 2*). The overall risk perception score was high (mean 4.84 on a 1–7 scale, with 1 = strongly disagree, 7 = strongly agree), with nurses reporting significantly higher perceived risk (mean 4.98) than physicians (mean 4.68), t(281) = −2.546, *p* < 0.05.

Significant group differences were also observed regarding specific risks. A greater proportion of nurses (39.8%) than physicians (18.2%) agreed that WhatsApp use interferes with workflow and may lead to errors (α = 0.000). Similarly, 60.3% of nurses compared to 50% of physicians expressed concern over using WhatsApp in the absence of clear guidelines (α = 0.015).

Regarding preferences for secure alternatives, 64.9% of nurses and 53.1% of physicians favored using a hospital-acquired secure app over WhatsApp for transmitting identifiable data (α = 0.005).

Statistical analysis also revealed significant gender-based differences in risk perception, with women reporting higher levels of concern (t(281) = −4.142, *p* < 0.05). No significant differences were found by profession within gender groups, indicating that observed disparities were likely due to gender composition (75% of nurses were women, compared to a near-even gender distribution among physicians).

Moreover, 88.7% of participants reported securing their smartphones with a password, facial recognition, or fingerprint. Approximately 51% used more than one security measure.

### Predictors of whatsapp use – regression analysis

A hierarchical linear regression was conducted to identify predictors of WhatsApp use. In the first step, demographic background variables were entered; in the second, key predictor variables were added. As shown in Table 2, the overall model was statistically significant, *F(*9,267) = 26.092, *p* < 0.01, explaining 46.8% of the variance in WhatsApp use.

**Table 2:** Regression coefficients for predicting WhatsApp use among medical and nursing staff in general hospitals

Predictors	β	SE	B	T	*R*²
**Step 1**					
Gender	−0.070	0.092	−0.103	−1.116	
Profession	0.077	0.092	0.110	1.192	
Age group	−0.112	0.042	−0.076	−1.816	
Medical wing	−0.065	0.035	−0.038	−1.066	
Hospital ownership	0.008	0.134	0.019	0.139	
Cumulative *R²*					**0.028**
**Step 2**					
Gender	−0.088	0.071	−0.128	−1.811	
Profession	0.025	0.073	0.036	0.495	
Age group	−0.170	0.032	−0.116	−3.589**	
Medical wing	0.005	0.027	0.003	0.109	
Hospital ownership	−0.035	0.102	−0.077	−0.759	
Personal benefit	0.516	0.036	0.352	9.680**	
Regulatory organizational benefit	0.156	0.034	0.112	3.292**	
Normative organizational benefit	0.236	0.038	0.160	4.259**	
Risk	−0.076	0.034	−0.054	−1.578	
Cumulative *R²*					**0.468**

*p < 0.05, **p < 0.01

Four variables emerged as significant predictors: age group, perceived personal utility, perceived regulatory utility, and perceived normative utility. A negative correlation was found between age and WhatsApp use, indicating that younger participants were more likely to use the app. At the same time, higher levels of all three utility types were positively associated with increased WhatsApp use.

Given the significant gender difference in risk perception, a separate regression model was conducted for female participants. In this model, four predictors were significant: age group, personal utility, normative utility, and perceived risk. Perceived risk showed a negative association with usage – indicating that, among women, risk considerations significantly influenced WhatsApp professional usage.

**Fig. 1:**
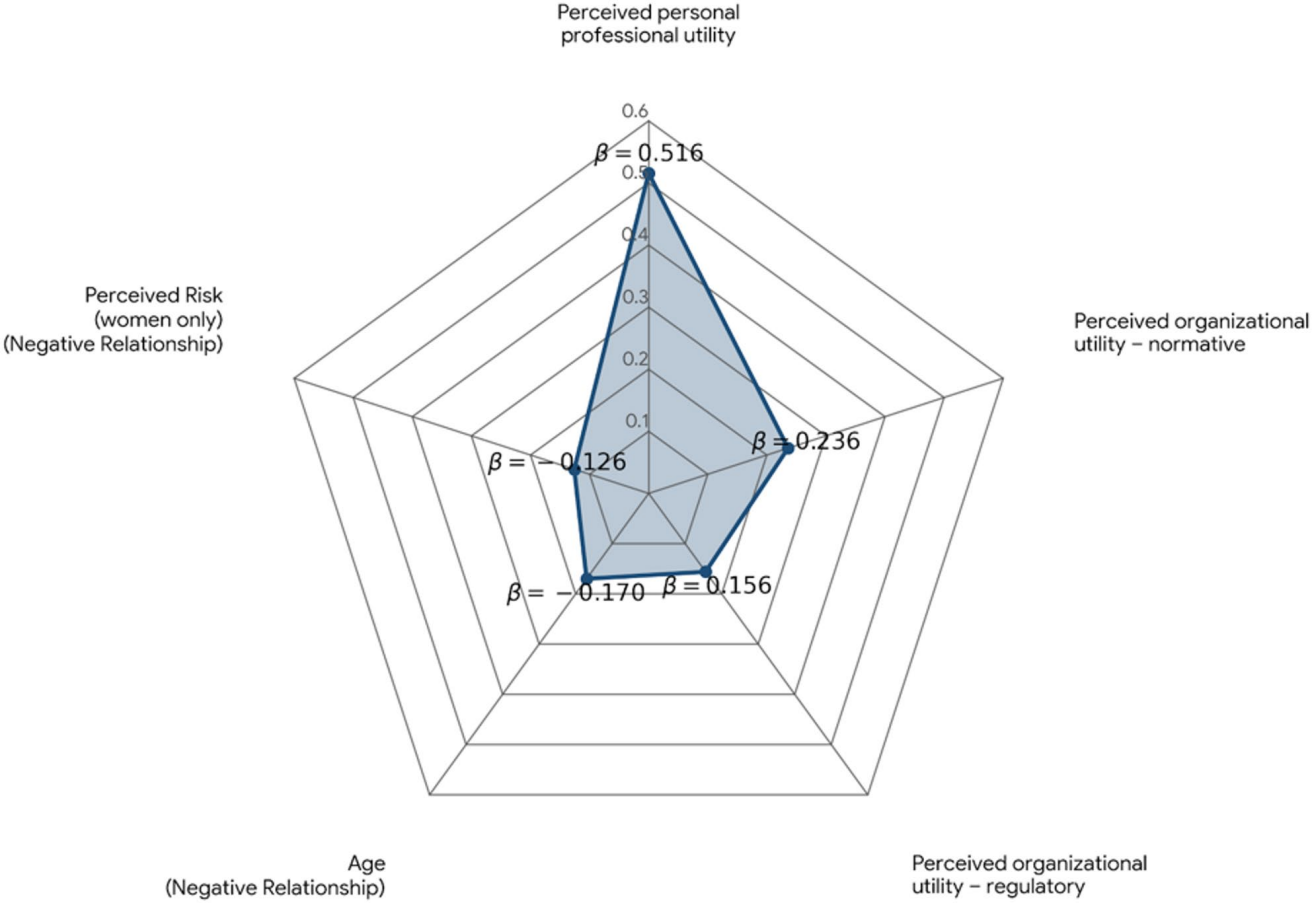
Predictors of WhatsApp use among medical and nursing staff in Israeli hospitals

Figure 1 summarizes and illustrates the relative weight of the main predictors of WhatsApp use identified in this study.

## Discussion

The findings of this study confirm that WhatsApp has become a central component of daily communication among medical and nursing staff in participating in our survey, mirroring global patterns of informal digital adoption within healthcare systems [2, 10, 29]. This “backdoor integration” of communication technology – widespread, unregulated, and largely self-organized – highlights the growing gap between technological practice and formal governance in clinical settings [29].

At the individual level, perceived personal benefit was the strongest driver of use. Participants emphasized time savings, smoother inter-professional communication, and the platform’s constant availability as primary advantages. These findings are consistent with prior research demonstrating WhatsApp’s contribution to communicative efficiency and improvement of clinical workflows [2]. Physicians, in particular, rely on WhatsApp, presumably due to the need for immediate consultation with specialists or senior on-call colleagues who are not physically present in the hospital – illustrating the application’s role as an adaptive tool that meets urgent clinical demands more efficiently than existing institutional systems.

At the organizational level, a clear asymmetry was found between normative and regulatory influence. Formal regulatory utility, reflecting adherence to official mandates, ranked lowest across all dimensions (mean = 1.86), underscoring policy ambiguity and the absence of coherent managerial guidance. In contrast, normative influence – reflected in the perception that “everyone uses WhatsApp” – proved decisive, aligning with recent literature showing that, when regulation lags behind reality, social norms become the dominant mechanism of professional governance [29]. The systematic disregard for privacy regulations raises fundamental questions about institutional integrity. As Scott [30] argues, professionals act as “institutional agents” who must navigate conflicting logics. In this case, a clash emerges between the regulatory logic (compliance with guidelines and privacy laws) and the professional logic of clinical efficiency and immediate patient care. The findings suggest that under conditions of “resource constraints” – whether in Israel or Italy – where institutional systems are slow or inaccessible, frontline workers adopt WhatsApp not as an act of rebellion, but as a calculated “survival strategy” to maintain operational continuity. This creates a state of “normalized deviance,” where the normative force of the law erodes because strict adherence is perceived as a hindrance to the primary professional goal: caring for the patient. Thus, non-compliance becomes rationalized as a necessary trade-off for systemic efficiency.

“Crucially, this prioritization of efficiency occurs even when privacy preservation is perceived by the staff as a core professional value, rather than merely an external mandate. This internal dissonance was particularly striking in the sharing of identifiable information. Although most respondents stated that such sharing should be avoided, over three-quarters of medical staff and more than half of nursing staff admitted to having done so. Similar to prior studies, this suggests that the perceived efficiency of WhatsApp outweighs abstract concerns about privacy protection [2, 10, 16]. In practice, confidentiality violations are not exceptional but normalized within routine communication – raising ethical and legal concerns about patient privacy and institutional liability.

Overall risk perception was high (mean = 4.84), in line with the findings of De Benedictis et al. [29]. Yet, its deterrent effect was partial and gender-specific. Women – predominantly nurses – reported higher risk perception and correspondingly lower use, consistent with behavioral research showing greater professional risk aversion among women [36].

This sensitivity positions nursing staff as potential agents of cultural change, capable of advancing responsible communication norms and influencing policy implementation from within clinical teams [37].

Age also emerged as a significant determinant: younger participants reported higher levels of use, consistent with global patterns of technology adoption and digital literacy [38]. As younger generations continue to enter the healthcare workforce, WhatsApp use is expected to increase, reinforcing the need for formal regulation.

When viewed comparatively, our findings align closely with the earlier Italian study by De Benedictis et al. [29], despite contextual and temporal differences. Both samples were predominantly female (67% in the Italian study; 62.5% in the present one), but the Israeli sample was relatively younger – only 52% of participants were over age 40, compared to 65% in the Italian cohort. This difference in age distribution may reflect demographic changes and workforce trends in healthcare systems over time.

Both studies revealed the same structural dynamics: ubiquitous use of WhatsApp for both personal and professional communication, stronger normative than regulatory influences, and an inverse relationship between perceived risk and actual use. The persistence of these findings over six years underscores that technical improvements in encryption and data protection have not resolved the underlying ethical and organizational tensions surrounding mobile messaging in healthcare.

### Strengths and Limitations

This study offers several important strengths. First, the sample size was relatively large (*N* = 281) and included both nursing and medical staff from multiple hospitals, allowing for meaningful subgroup analyses and enhancing external validity. The use of both digital and paper-based questionnaires, and data collection across different institutional contexts, improved accessibility and reduced selection bias.

Second, reliability analysis of the questionnaire showed high internal consistency for most variables (Cronbach’s alpha > 0.7), with the ‘Policy’ construct slightly below the threshold (α = 0.679), which is considered acceptable for exploratory adaptations, particularly given the small number of items (*n* = 4) comprising this sub-scale. This suggests strong coherence among items and reinforces the validity of the multidimensional framework used to assess personal, organizational, normative, and risk-related perceptions.

The study also benefited from a comprehensive conceptual design, evaluating both drivers and deterrents of WhatsApp use, including personal benefit, organizational influence (normative and regulatory), and perceived risk.

Nevertheless, several limitations should be acknowledged. The use of convenience sampling may limit representativeness. While this limitation cannot be fully eliminated, it was partially addressed by recruiting participants through diverse professional networks and communication channels, both digital and paper-based, to enhance heterogeneity and reduce selection bias. As a concentration of responses was detected from one hospital, a one-way ANOVA was conducted to compare three respondent groups: printed-form respondents (29%), nationwide digital respondents (30%), and a concentration of digital respondents from one hospital (41%). Among nursing staff, no significant differences were found across these groups, indicating internal stability of responses. Among medical staff, only two variables – perceived risk and normative utility – differed significantly, with higher scores among respondents from hospitals other than the one with the local concentration. This indicates some contextual variation while still supporting the overall robustness of the findings.

Also, due to the viral distribution of the survey via social media groups (mainly WhatsApp and Facebook), the total number of potential respondents exposed to the link is unknown. Therefore, an exact response rate could not be calculated, and it is not possible to assess non-response bias.

Additionally, self-reported data is subject to social desirability bias, particularly concerning the transmission of sensitive patient information. There were also demographic imbalances between groups, such as a gender skew in the nursing group and a younger profile among medical staff, which may affect generalizability, though they reflect real-world workforce patterns in Israeli hospitals.

## Conclusions and policy recommendations

Overall, this study reinforces that WhatsApp’s prevalence is not a technological accident but a systemic adaptation to unmet communicative needs. Its success reflects a mismatch between clinicians’ everyday realities and the institutional tools and policies available to them. The challenge for healthcare systems is therefore cultural and organizational: to reconcile the need for rapid, reliable communication with the imperatives of confidentiality, professionalism, and trust. Achieving this balance requires pragmatic, evidence-based regulation that acknowledges current practice while guiding it toward secure, ethically aligned use.

A blanket prohibition on the use of WhatsApp would be impractical and likely unenforceable, given the application’s widespread integration into daily practice. Instead, the development of practical guidelines that enable secure use of the platform – while maximizing its benefits and minimizing potential harms – is recommended. These guidelines should address key issues such as permissible and prohibited types of data sharing, the proper use of features like message deletion and end-to-end encryption, and minimum-security standards for mobile devices.

In this regard, the regulatory approach adopted by the United Kingdom’s National Health Service (NHS) offers a valuable model. The NHS’s official guidance on *Using Mobile Messaging in Health and Care Settings* explicitly permits the use of instant messaging applications when clinically necessary but under strict safeguards: minimizing personal data shared, ensuring device-level security (e.g., password protection, remote wipe), and transferring any clinical decisions communicated via mobile messaging into the formal health record as soon as possible [39].

Although the position paper published by the Israeli Privacy Protection Authority in March 2024 represents a significant first step in addressing these challenges [28] it lacks sufficient operational clarity and fails to provide detailed guidance suited to the clinical realities of hospital work. The regulatory authorities must therefore take the lead in formulating applicable and enforceable recommendations, while individual healthcare institutions should concurrently develop their own detailed internal policies adapted to their specific needs and operational context.

From a technological perspective, healthcare institutions should explore the possibility of acquiring secure, institutionally-managed messaging platforms that offer both robust data protection and user-friendly interfaces. This direction aligns with the regulatory and accreditation frameworks established by U.S. health oversight bodies – including the Centers for Medicare & Medicaid Services (CMS) [40], and The Joint Commission (TJC) [41], the national accreditation body for healthcare organizations. Both permit clinical texting between care team members only through HIPAA-compliant Secure Texting Platforms (STPs), which must ensure encryption, identity verification, audit logging, integration with the patient’s electronic health record, and full institutional control over data storage and deletion.

However, technological solutions alone will not suffice. Sustainable change will require a coordinated strategy that incorporates education, organizational culture, technological tools, and appropriate regulations. In parallel with policy development, there is a need to invest in broad-based training programs to enhance awareness of information security and privacy obligations. Particular attention should be paid to medical staff, who were found in this study to participate less in data security training and to be more likely to share identifiable information via WhatsApp. Gender-based strategies may also be beneficial, as the study identified a higher perception of risk among women, and especially among the predominantly female nursing staff. These groups may serve as natural entry points for behavioral change and institutional adaptation.

Finally, there is a pressing need for the development of ongoing monitoring mechanisms to evaluate how instant messaging tools are being used in practice and to assess the effectiveness of emerging policies over time. A flexible regulatory framework is essential – one that can evolve in response to technological advances, support innovation in clinical communication, and ensure that high standards of data security are maintained.

The central challenge facing policymakers is to identify and maintain a delicate equilibrium: one that allows clinical teams to benefit from the advantages of modern communication technologies while mitigating the inherent risks. A regulatory approach that acknowledges the widespread reality of WhatsApp use and offers realistic, implementable solutions is likely to be far more effective than rigid prohibitions that cannot be meaningfully enforced.

## Supplementary Information

Supplementary Material 1

Supplementary Material 2

## Data Availability

The datasets generated and/or analyzed during the current study are available from the corresponding author on reasonable request.

## References

[1] About WhatsApp [Internet]. 2025 [cited 2025 Jun 11]. Available from: https://www.whatsapp.com/about

[2] Shaarani I, El-Kantar A, Hamzeh N, Jounblat M, El-Yaman T, Habanjar M, et al. Interprofessional communication of physicians using whatsapp: Physicians’ perspective. Telemed e-Health. 2020;26(10):1257–64. 10.1089/tmj.2019.0216 32083515

[3] Chan WSY, Leung AYM. Use of social network sites for communication among health professionals: Systematic review. J Med Internet Res. 2018;20:e117. 29592845 10.2196/jmir.8382 PMC5895921

[4] Westgarth D. Has the pandemic changed the way we communicate? BDJ Pract. 2021;34(8):8–14.

[5] Gebbia V, Piazza D, Valerio MR, Firenze A. WhatsApp messenger use in oncology: a narrative review on pros and contras of a flexible and practical, non-specific communication tool. Ecancermedicalscience. 2021;15:1334. 35211203 10.3332/ecancer.2021.1334 PMC8816506

[6] Giordano V, Koch H, Godoy-Santos A, Dias Belangero W, Esteves Santos Pires R, Labronici P. WhatsApp messenger as an adjunctive tool for telemedicine: an overview. Interact J Med Res. 2017;6(2):e11. 28733273 10.2196/ijmr.6214 PMC5544893

[7] Johnston MJ, King D, Arora S, Behar N, Athanasiou T, Sevdalis N, et al. Smartphones let surgeons know WhatsApp: An analysis of communication in emergency surgical teams. Am J Surg. 2015;209(1):45–51. 25454952 10.1016/j.amjsurg.2014.08.030

[8] Liu X, Sutton PR, McKenna R, Sinanan MN, Fellner BJ, Leu MG, et al. Evaluation of secure messaging applications for a health care system: a case study. Appl Clin Inform. 2019;10(1):140–50. 30812040 10.1055/s-0039-1678607 PMC6393161

[9] Howe J, Magazine W. Impact of mobile tablet conputers on internai medicine resident efficiency. Wired. 2008;172(September):436–8. 10.1001/archinternmed.2012.45 22412110

[10] Nikolic A, Wickramasinghe N, Claydon-Platt D, Balakrishnan V, Smart P. The Use of Communication Apps by Medical Staff in the Australian Health Care System: Survey Sudy on Prevalence and Use. JMIR Med Inf. 2018;6(1):e9. 10.2196/medinform.9526 PMC5889814 29426813

[11] Tran K, Morra D, Lo V, Quan S, Wu R. The use of smartphones on General Internal Medicine wards: a mixed methods study. Appl Clin Inform. 2014;5(3):814–23. 25298819 10.4338/ACI-2014-02-RA-0011 PMC4187096

[12] Frizzell JD, Ahmed B. Text Messaging Versus Paging New Technology for the Next Generation [Internet]. Available from: http://www.jointcommission.org/assets/1/18/Root_ 10.1016/j.jacc.2014.11.001 25524347

[13] de Jong A, Donelle L, Kerr M. Nurses’ use of personal smartphone technology in the workplace: Scoping review. JMIR Mhealth Uhealth. 2020;8(11):e18774. 33242012 10.2196/18774 PMC7728531

[14] Prochaska MT, Bird AN, Chadaga A, Arora VM. Resident use of text messaging for patient care: Ease of use or breach of privacy? JMIR Med Inf. 2015;3(4):e37. 10.2196/medinform.4797 PMC6858010 26611620

[15] Knapova L, Kruzikova A, Dedkova L, Smahel D. Who Is Smart with Their Smartphones? Determinants of Smartphone Security Behavior. Cyberpsychol Behav Soc Netw. 2021;24(9):584–92. 34152852 10.1089/cyber.2020.0599

[16] John B, McCreary C, Roberts A. Smartphone technology for communications between clinicians – A scoping review. J Dent. 2022;122:104112. 35413411 10.1016/j.jdent.2022.104112

[17] GDPR Regulation [Internet]. Nextep Group. 2025 [cited 2025 Sep 13]. Available from: https://www.nextep.co.il/gdpr-lp/?gclid=Cj0KCQjwgO2XBhCaARIsANrW2X1_jioi6QRnjKO4xKI8t7DxPsUgJjVFQm_QB5gOv4x4ibMkYjBueAsaAkPQEALw_wcB

[18] Hewitt B, Dolezel D, McLeod A. Mobile Device Security: Perspectives of Future Healthcare Workers. Perspect Heal Inf Manag. 2017;14(Winter):1c. PMC5430111 28566992

[19] Social Media and Online Services Usage in Israel. 2025 Data [Internet]. Israel Internet Association. Available from: https://www.isoc.org.il/sts-data/social-media-usage-survey-2025

[20] Barayev E, Shental O, Yaari D, Zloczower E, Shemesh I, Shapiro M. WhatsApp tele-medicine – usage patterns and physicians views on the platform. Isr J Health Policy Res. 2021;8:1–9. 10.1186/s13584-021-00468-8 PMC8167384 34074319

[21] Basic Law: Human Dignity and Freedom. Basic laws of Israel Israel. 1992 p. 7–8.

[22] Privacy Protection Law. Statute Book Israel; 1981.

[23] Ministry of Health. Patient’s Rights Law. Israel Israel; 1996.

[24] Bar Siman Tov M. Ethical Code for the Protection of Medical Confidentiality and Personal Information Privacy in the Healthcare System [Internet]. Jerusalem; 2020. Available from: https://www.health.gov.il/hozer/mk02_2020.pdf

[25] JCI. Staff Qualifications and Education (SQE). In: Joint Commission internationa, editor. Joint Commission International Accreditation Standards for Hospitals: Including Standards for Academic Medical Center Hospitals. 7th ed. Oak Brook, Illinois: JCR Publishing; 2020. p. 276–7.

[26] Basic regulation for cyber protection in the Israeli healthcare system. Director General Circular, Ministry of Health Israel; 2022.

[27] Protecting patient privacy in the provision of remote medical services [Internet]. Ministry of Justice, Israeli Privacy Protection Authority. 2022. Available from: https://www.gov.il/he/pages/medical_information_share

[28] Protecting Patient Privacy in the Digital Transfer of Medical Information [Internet]. Jerusalem. 2024. Available from: https://www.gov.il/he/pages/medical_information_share

[29] Benedictis A, De, Lettieri E, Masella C, Gastaldi L, Macchini G, Santu C, et al. WhatsApp in hospital? An empirical investigation of individual and organizational determinants to use. PLoS ONE. 2019;14(1):e0209873. 30633754 10.1371/journal.pone.0209873 PMC6329505

[30] Moore GC, Benbasat I. Development of an instrument to measure the perceptions of adopting an information technology innovation. Inf Syst Res. 1991;2(3):192–222.

[31] Davis FD, Perceived, Usefulness. Perceived Ease of Use, and User Acceptance of Information Technology. MIS Q [Internet]. 1989;13(3):319. Available from: https://www.jstor.org/stable/249008?origin=crossref

[32] Scott WR. Lords of the dance: professionals as institutional agents. Organ Stud. 2008;29(2):219–38.

[33] Ministry of Health. Human Resources in Health Professions 2022. Jerusalem; 2023.

[34] Salary Expenditures in the Public. Healthcare System for 2020. Dep Wages Labor Agreements; 2021.

[35] Health professions workforce 2020. Inf Dep Med Technol Div Minist Heal [Internet]. 2021; Available from: www.health.gov.il/moh-info

[36] Hurley D, Choudhary A. Role of gender and corporate risk taking. Corp Gov. 2020;20(3):383–99.

[37] Hajizadeh A, Zamanzadeh V, Kakemam E, Bahreini R, Khodayari-Zarnaq R. Factors influencing nurses participation in the health policy-making process: a systematic review. BMC Nurs. 2021;20(1):1–9. 34253210 10.1186/s12912-021-00648-6 PMC8273557

[38] Magsamen-conrad K, Muhleman J. Mobile technology adoption across the lifespan: a mixed methods investigation to clarify adoption stages, and the influence of diffusion attributes. Comput Human Behav. 2020;112:106456. 32834465 10.1016/j.chb.2020.106456 PMC7305511

[39] Texting. emailing and messaging patients and service users [Internet]. NHS. Available from: https://transform.england.nhs.uk/information-governance/guidance/use-mobile-messaging-software-health-and-care-settings/?utm_source=chatgpt.com

[40] Centers for Medicare and Medicaid Services. Texting of Patient Information and Orders for Hospitals and CAHs [Internet]. 2024 [cited 2025 Oct 14]. Available from: https://www.cms.gov/medicare/health-safety-standards/quality-safety-oversight-general-information/policy-memos-states-and-cms-locations/texting-patient-information-and-orders-hospitals-and-cahs?utm_source=chatgpt.com

[41] The Joint Commission. Use of secure text messaging for patient information and orders [Internet]. Joint Commission Online. Available from: https://www.jointcommission.org/en-us/knowledge-library/newsletters/joint-commission-online/05-jun-24

