# Supplementary material for: When practice outpaces policy: Whatsapp use among nursing and medical staff in Israeli hospitals
Source: Isr J Health Policy Res. 2026 Apr 2;15:8. doi: 10.1186/s13584-026-00754-3 (PMC13045068; doi:10.1186/s13584-026-00754-3)
Supplement: Supplementary file 1 — Supplementary Material 1 [file 13584_2026_754_MOESM1_ESM.docx]

# **Appendix 1 – Translated Questionnaire (Hebrew to English)**

## Introduction

Dear Participant,
We kindly invite you to take part in a research study aiming to map the scope and characteristics of WhatsApp use for communication among medical and nursing staff in Israeli hospitals.

This study is part of a graduate thesis conducted at the School of Public Health, University of Haifa.
Completing the questionnaire should take no more than 10 minutes.
Participation is voluntary, and submission of the questionnaire constitutes informed consent.
The questionnaire is anonymous and contains no identifying information.
The questions are phrased in masculine grammatical form for convenience but refer to all genders.
The questionnaire is intended for hospital staff only.

Thank you for your cooperation,
Drora Ben Michael Winker and Dr. Maya Peled Raz
School of Public Health, University of Haifa
For inquiries, please contact: drorab@hymc.gov.il

## Section A: General Information

1. What type of hospital do you work in?
☐ General ☐ Geriatric ☐ Psychiatric ☐ Other: ___________

2. The hospital I work in is:
☐ Governmental / Municipal ☐ Affiliated with Clalit or Maccabi ☐ Other: ___________

3. Age group:
☐ 21–30 ☐ 31–40 ☐ 41–50 ☐ 51 and above

4. Gender:
☐ Female ☐ Male ☐ Other

5. Profession:
☐ Physician (skip to question 7) ☐ Nurse (continue to question 6)

6. Nursing Role (select all that apply):
☐ Head nurse / Deputy head nurse
☐ Team leader / Clinical instructor
☐ Regular staff nurse
☐ Administrative / Coordinating role

7. Medical Role:
☐ Head of department / Deputy / Unit manager / Service director
☐ Specialist
☐ Resident
☐ Intern

8. Years of professional experience:
☐ 0–5 ☐ 6–10 ☐ 11–20 ☐ 21–30 ☐ More than 30

9. Department:
☐ Internal medicine / Pediatrics (including ER)
☐ Surgical unit (including OR)
☐ Obstetrics and gynecology
☐ Psychiatry
☐ Geriatrics
☐ Other: ___________

## Section B: Use of WhatsApp – Personal Purposes

Instruction: Please indicate how frequently you use WhatsApp for the following personal activities.

Scale: 1 = Never 2 = Rarely 3 = Sometimes 4 = Often 5 = Always (All the time)

10. I use WhatsApp for one-on-one personal communication

11. I use WhatsApp to communicate in group chats

12. I use WhatsApp to share moments from my personal life with others

13. I use WhatsApp to send written messages

14. I use WhatsApp to send images

15. I use WhatsApp to send voice messages

16. I use WhatsApp to send videos

17. I use WhatsApp in the presence of other people

18. I connect to WhatsApp many times a day **for personal purposes**

## Section B: Use of WhatsApp – Professional Purposes

Instruction: Please indicate how frequently you use WhatsApp for the following work-related activities.

Scale: 1 = Never 2 = Rarely 3 = Sometimes 4 = Often 5 = Always

19. I use WhatsApp to share scientific or professional information with colleagues

20. I use WhatsApp to discuss clinical cases with the team **without mentioning identifiable patient information**

21. I use WhatsApp to request or provide professional guidance within the team **without identifying patient information**

22. I use WhatsApp to share clinical images or videos with the team

23 I use WhatsApp to request information or receive or give instructions to staff members **while using identifying information** of the patient. I use

24. Since the COVID-19 pandemic, I use WhatsApp to share **identifiable patient information**

## Section C: Perceived Usefulness – Individual Perspective

Instruction: Please indicate the extent to which you agree or disagree with the following statements regarding the use of WhatsApp for professional purposes.

Scale: 1 = Strongly disagree 2 = Disagree 3 = Slightly disagree 4 = Neither agree nor disagree 5 = Slightly agree 6 = Agree 7 = Strongly agree

25. I am convinced that using WhatsApp improves communication among healthcare staff

26. Using WhatsApp saves working time because it is faster than phone calls or email

27. I am convinced that if everyone used WhatsApp, there would be greater and more effective sharing of clinical knowledge

28. Using WhatsApp can greatly contribute to reducing hospital costs

29. Using WhatsApp at work decreases my productivity (e.g., I get distracted by non-work-related matters)

30. Using WhatsApp has a positive impact on my research activity (e.g., easier to share data and results)

31. Using WhatsApp positively affects my teaching activity

32. Using WhatsApp for communication among staff increases workload

33. Using WhatsApp for communication among clinicians improves continuity of care

34. WhatsApp is preferable to other messaging apps due to its widespread use

35. I support the use of my personal smartphone for work-related purposes

## Section D: Perceived Usefulness – Organizational/Regulative Perspective

Instruction: Please indicate how often the following requests or instructions were made by hospital management regarding WhatsApp use.

Scale: 1 = Never 2 = Rarely 3 = Sometimes 4 = Often 5 = Always

36. The hospital management has asked me not to use WhatsApp among colleagues

37. The hospital management has asked me not to share **identifiable patient information** via WhatsApp

38. The hospital management requires me to use WhatsApp

39. During the COVID-19 pandemic, the hospital management required me to use WhatsApp

## Section E: Normative Factors

Instruction: Please indicate your level of agreement with the following statements regarding the use of WhatsApp among your colleagues.

Scale: 1 = Strongly disagree 2 = Disagree 3 = Slightly disagree 4 = Neither agree nor disagree 5 = Slightly agree 6 = Agree 7 = Strongly agree

40. My colleagues use WhatsApp for **personal purposes**

41. My colleagues use WhatsApp for **professional purposes**

42. My colleagues use WhatsApp to share scientific information

43. My colleagues use WhatsApp to share patient-related information

44. My colleagues do not want to use WhatsApp for professional purposes

## Section F: Perceived Risks

Instruction: Please indicate your level of agreement with the following statements regarding potential risks associated with the use of WhatsApp in clinical settings.

Scale: 1 = Strongly disagree 2 = Disagree 3 = Slightly disagree 4 = Neither agree nor disagree 5 = Slightly agree 6 = Agree 7 = Strongly agree

45. Transmitting patient information via WhatsApp is safe and does not involve risks

46. Sending clinical data via WhatsApp involves legal risks for healthcare professionals

47. Using WhatsApp involves risks to patient privacy, medical confidentiality, and data protection

48. Using WhatsApp entails the risk of uncontrolled dissemination of sensitive information

49. There are no clear guidelines in the team on the secure use of WhatsApp

50. Using WhatsApp during clinical work distracts staff and increases the risk of errors

51. WhatsApp does not pose a confidentiality risk because data is end-to-end encrypted

52. WhatsApp does not pose a confidentiality risk because my phone is protected by password, fingerprint, or facial recognition

53. WhatsApp compromises medical confidentiality because images are automatically saved to the device gallery and cloud

54. Using WhatsApp constitutes a breach of patient confidentiality

55. I am concerned about the risks associated with using WhatsApp

56. It is important to ensure that only **de-identified patient** information is transmitted or received

57**. Identifiable patient** data received via WhatsApp should be deleted immediately

58. I prefer to use a secure hospital-approved messaging app for sharing sensitive patient information

59. If asked, I would be willing to install a secure, hospital-approved messaging app on my personal smartphone

## Section G: Actual Information Shared via WhatsApp

60. During the past year, which of the following types of content have you sent or received via WhatsApp as part of your work? (You may select more than one option):

☐ Patient labels

☐ Questions addressed to colleagues

☐ Responses to colleagues' requests

☐ Work-related images (e.g., wounds)

☐ Clinical reports

☐ Imaging results

☐ ECG recordings

☐ Pathology reports

☐ Surgery reports

☐ Laboratory test results

☐ Administrative matters (e.g., test approvals, procedures, guidelines)

☐ Reports to management

## Section H: Information Security and Guidelines

61. Have you received information security training during the past year?
☐ Yes ☐ No ☐ Don’t know

62. Does your hospital have a protocol regarding the use of WhatsApp?
☐ Yes ☐ No ☐ Don’t know

63. Does your hospital have a secure instant messaging application?
☐ Yes ☐ No ☐ Don’t know

64. (If you answered “Yes” to question 63): Is the secure app commonly used in your hospital?
☐ Yes ☐ No ☐ Don’t know

65. (If you answered “No” to question 63): In your opinion, what are the reasons the secure app is not commonly used? (You may select more than one option)
☐ Staff are reluctant to install the app on their personal phones
☐ The number of users is limited due to high licensing costs
☐ The app requires password login, which is inconvenient
☐ It is difficult to share images or videos – the interface is not user-friendly
☐ WhatsApp is much more convenient and accessible
☐ Other: ___________

66. My smartphone is protected by (You may select more than one option):
☐ Password
☐ Fingerprint
☐ Face recognition

☐ Not protected

## Notes

- Section B (Professional Use), Item 23: No equivalent in the Italian questionnaire; added to reflect COVID-specific data sharing.
- Section C, Items 33–34: Not present in Italian version; relate to app popularity and personal smartphone use.
- Section D, Items 37–38: Unique to Israeli context; Italian tool does not include management enforcement of WhatsApp.
- Section F, Items 50–52: Focus on encryption and smartphone-level security; these are not in the Italian version.
- Section H (Entire): These items (Q60–64) are unique to this version and were not included in the original Italian questionnaire. They provide context-specific insight into barriers to secure messaging use.
