# Supplementary material for: When practice outpaces policy: Whatsapp use among nursing and medical staff in Israeli hospitals
Source: Isr J Health Policy Res. 2026 Apr 2;15:8. doi: 10.1186/s13584-026-00754-3 (PMC13045068; doi:10.1186/s13584-026-00754-3)
Supplement: Supplementary file 2 — Supplementary Material 2 [file 13584_2026_754_MOESM2_ESM.docx]

**Appendix 2**

**Table S1: Professional Use of WhatsApp** (Statement 19-24)

| **Statement** | **Staff** | **Never** | **Rarely** | **Sometimes** | **Often** | **Always** | **M (SD)** |
| --- | --- | --- | --- | --- | --- | --- | --- |
| I use WhatsApp to share scientific information with peers | Nurses* | 4 (2.7%) | 23 (15.3%) | 35 (23.3%) | 53 (35.3%) | 35 (23.3%) | 3.61 (1.086) |
|  | Physicians** | 3 (2.3%) | 9 (6.9%) | 47 (35.9%) | 40 (30.5%) | 32 (24.4%) | 3.68 (0.994) |
| I use WhatsApp with the team to discuss clinical situations **without** mentioning **patient identifying information** | Nurses | 28 (18.5%) | 26 (17.2%) | 38 (25.2%) | 42 (27.8%) | 17 (11.3%) | 2.96 (1.285) |
|  | Physicians | 4 (3.0%) | 14 (10.6%) | 54  (40.2%) | 39 (29.5%) | 22 (16.7%) | 3.46 (0.992) |
| I use WhatsApp to request information or receive/give instructions to staff **without** mentioning **patient identifying information** | Nurses | 16 (10.6%) | 25 (16.6%) | 26  (17.2%) | 55 (36.4%) | 29 (19.2%) | 3.37 (1.263) |
|  | Physicians | 11 (8.3%) | 21 (15.9%) | 36  (27.3%) | 39 (29.5%) | 25 (18.9%) | 3.35 (1.198) |
| I use WhatsApp to receive or transmit clinical information about patients in the form of photos or videos | Nurses | 34 (22.5%) | 32 (21.2%) | 36  (23.8%) | 33 (21.9%) | 16 (10.6%) | 2.77 (1.309) |
|  | Physicians | 14 (10.6%) | 30 (22.7%) | 43  (32.6%) | 27 (20.5%) | 18 (13.6%) | 3.04 (1.188) |
| I use WhatsApp to request information or receive/give instructions to staff **using patient identifying information** | Nurses | 62 (41.1%) | 31 (20.5%) | 27  (17.9%) | 19 (12.6%) | 12 (7.9%) | 2.26 (1.324) |
|  | Physicians | 30 (22.7%) | 37 (28.0%) | 31  (23.5%) | 23 (17.4%) | 11 (8.3%) | 2.61 (1.246) |
| During the COVID-19 pandemic, my WhatsApp use included **identifiable patient information** | Nurses | 83 (55.0%) | 25 (16.6%) | 20  (13.2%) | 15 (9.9%) | 8 (5.3%) | 1.94 (1.250) |
|  | Physicians** | 56 (43.0%) | 30 (23.1%) | 21  (16.2%) | 17 (13.1%) | 6 (4.6%) | 2.13 (1.235) |

*1 missing, ** 2 missing

**Table S2: Perceived Professional Personal Utility** (Statement 25-35)

| **Statement** | **Staff** | **Strongly Disagree** | **Disagree** | **Somewhat Disagree** | **Neither Agree nor Disagree** | **Somewhat Agree** | **Agree** | **Strongly Agree** | **M (SD)** |
| --- | --- | --- | --- | --- | --- | --- | --- | --- | --- |
| I am convinced that WhatsApp use improves communication between caregivers | Nurses | 7  (4.6%) | 8  (5.3%) | 8  (5.3%) | 20 (13.2%) | 27  (17.9%) | 33 (21.9%) | 48 (31.8%) | 5.27 (1.732) |
|  | Physicians | 2  (1.5%) | 1  (0.8%) | 5  (3.8%) | 13  (9.8%) | 30  (22.7%) | 31 (23.5%) | 50 (37.9%) | 5.73 (1.336) |
| I am convinced that WhatsApp use saves work time because it is faster than phone or email | Nurses | 6  (4.0%) | 4  (2.6%) | 9  (6.0%) | 17 (11.3%) | 28  (18.3%) | 34 (22.3%) | 53 (35.1%) | 5.46 (1.632) |
|  | Physicians | 2  (1.5%) | 2  (1.5%) | 1  (0.8%) | 12  (9.1%) | 23  (17.4%) | 38 (28.8%) | 54 (40.9%) | 5.89 (1.28) |
| I am convinced that if everyone used WhatsApp there would be broader and more efficient sharing of clinical knowledge | Nurses | 7  (4.6%) | 10  (6.6%) | 7  (4.6%) | 29 (19.2%) | 31  (20.5%) | 31 (20.5%) | 36 (23.8%) | 5.01 (1.701) |
|  | Physicians | 0  (0%) | 4  (3.0%) | 6  (4.5%) | 31 (23.5%) | 29  (22.0%) | 24 (18.2%) | 38 (28.8%) | 5.34 (1.386) |
| I am convinced that WhatsApp use can contribute greatly to reducing hospital costs | Nurses | 5  (3.3%) | 10  (6.6%) | 8  (5.3%) | 45 (29.8%) | 24  (15.9%) | 28 (18.5%) | 31 (20.5%) | 4.86 (1.625) |
|  | Physicians | 3  (2.3%) | 6  (4.5%) | 10 (7.6%) | 50 (38.0%) | 20  (15.2%) | 17 (12.9%) | 26 (19.7%) | 4.77 (1.528) |
| I am convinced that WhatsApp use at work reduces my productivity (my attention is distracted by factors unrelated to my work) | Nurses* | 17 (11.3%) | 30 (20.0%) | 21 (14.0%) | 27 (17.9%) | 31  (20.7%) | 19 (12.7%) | 5 (3.3%) | 3.68 (1.7) |
|  | Physicians | 15 (11.4%) | 22 (16.7%) | 35 (26.5%) | 26 (19.7%) | 20  (15.2%) | 7  (5.3%) | 7  (5.3%) | 3.48 (1.594) |
| I am convinced that WhatsApp use positively affects my research activities (easier to share data and results) | Nurses | 4  (2.6%) | 4  (2.6%) | 14 (9.3%) | 31 (20.5%) | 29  (19.2%) | 42 (27.7%) | 27 (17.9%) | 5.05 (1.498) |
|  | Physicians | 1  (0.8%) | 8  (6.1%) | 7  (5.3%) | 48 (36.4%) | 21  (15.9%) | 22 (16.7%) | 25 (18.9%) | 4.86 (1.471) |
| I am convinced that WhatsApp use positively affects my teaching activities | Nurses | 5  (3.3%) | 4  (2.6%) | 11 (7.3%) | 35 (23.2%) | 35  (23.2%) | 34 (22.5%) | 27 (17.9%) | 4.99 (1.499) |
|  | Physicians | 1  (0.8%) | 7  (5.3%) | 5  (3.8%) | 38 (28.8%) | 26  (19.8%) | 32 (24.2%) | 23 (17.4%) | 5.04 (1.411) |
| I am convinced that WhatsApp use for team communication increases workload | Nurses | 21 (13.9%) | 38 (25.2%) | 28 (18.5%) | 36 (23.8%) | 17  (11.3%) | 4  (2.6%) | 7  (4.6%) | 3.2 (1.566) |
|  | Physicians* | 12 (9.2%) | 35 (26.7%) | 28 (21.4%) | 27 (20.6%) | 16  (12.2%) | 8  (6.1%) | 5  (3.8%) | 3.34 (1.547) |
| I am convinced that WhatsApp use for communication between caregivers improves continuity of care | Nurses | 7  (4.6%) | 6  (4.0%) | 7  (4.6%) | 29 (19.2%) | 33  (21.9%) | 39 (25.8%) | 30 (19.9%) | 5.07 (1.594) |
|  | Physicians | 0  (0%) | 6  (4.5%) | 3  (2.3%) | 24 (18.2%) | 41  (31.1%) | 25 (18.9%) | 33 (25.0%) | 5.33 (1.334) |
| I am convinced that WhatsApp is preferable to other applications because of the app's widespread distribution | Nurses | 5  (3.3%) | 7  (4.6%) | 8  (5.3%) | 28 (18.5%) | 30  (19.9%) | 30 (19.9%) | 43 (28.5%) | 5.21 (1.63) |
|  | Physicians | 3  (2.3%) | 1  (0.8%) | 4  (3.0%) | 18 (13.6%) | 36  (27.3%) | 36 (27.3%) | 34 (25.8%) | 5.48 (1.333) |
| I support using my personal smartphone for work purposes | Nurses** | 10 (6.7%) | 11  (7.4%) | 12 (8.1%) | 21 (14.1%) | 27  (18.1%) | 37 (24.8%) | 31 (20.8%) | 4.87 (1.813) |
|  | Physicians | 5  (3.8%) | 4  (3.0%) | 6  (4.5%) | 12  (9.1%) | 31  (23.5%) | 44 (33.3%) | 30 (22.7%) | 5.36 (1.51) |

*1 missing, **2 missing

**Table S3: Perceived Organizational Benefit – Regulatory Factors** (Statements 36-39)

| **Statement** | **Staff** | **Not True** | **Rarely True** | **Sometimes** | **Often True** | **Always True** | **M (SD)** |
| --- | --- | --- | --- | --- | --- | --- | --- |
| Hospital management asks me not to use WhatsApp among colleagues | Nurses** | 85 (57.0%) | 19 (12.8%) | 21  (14.1%) | 17 (11.4%) | 7 (4.7%) | 1.94 (1.264) |
|  | Physicians | 105 (79.5%) | 15 (11.4%) | 8  (6.1%) | 2  (1.5%) | 2  (1.5%) | 1.34 (0.790) |
| Hospital management asks me not to transmit **identifiable patient data** via WhatsApp | Nurses** | 31 (20.7%) | 12 (8.0%) | 12  (8.0%) | 16 (10.7%) | 79 (52.7%) | 3.67 (1.641) |
|  | Physicians | 36 (27.3%) | 15 (11.4%) | 19  (14.4%) | 16 (12.1%) | 46 (34.8%) | 3.16 (1.648) |
| Hospital management forces me to use WhatsApp | Nurses** | 75 (50.3%) | 16 (10.7%) | 21  (14.1%) | 23 (15.4%) | 14 (9.4%) | 2.23 (1.438) |
|  | Physicians | 87 (65.9%) | 14 (10.6%) | 16  (12.1%) | 11 (8.3%) | 4  (3.0%) | 1.72 (1.148) |
| During the COVID-19 pandemic, hospital management forced me to use WhatsApp | Nurses** | 82 (55.0%) | 8  (5.4%) | 20  (13.4%) | 27 (18.1%) | 12 (8.1%) | 2.19 (1.454) |
|  | Physicians** | 91 (70.5%) | 9  (7.0%) | 16  (12.4%) | 10 (7.6%) | 3  (2.3%) | 1.64 (1.110) |

**2 missing

**Table S4: Perceived Organizational Benefit – Normative Factors**  (Statements 40-44)

| **Statement** | **Staff** | **Strongly Disagree** | **Disagree** | **Somewhat Disagree** | **Neither Agree nor Disagree** | **Somewhat Agree** | **Agree** | **Strongly Agree** | **M (SD)** |
| --- | --- | --- | --- | --- | --- | --- | --- | --- | --- |
| I am convinced that my colleagues use WhatsApp for personal purposes | Nurses* | 0  (0%) | 1  (0.7%) | 8  (5.3%) | 10  (6.7%) | 19  (12.7%) | 38 (25.3%) | 74 (49.3%) | 6.05 (1.217) |
|  | Physicians | 1  (0.8%) | 0  (0%) | 4  (3.0%) | 7  (5.3%) | 16  (12.1%) | 31 (23.5%) | 73 (55.3%) | 6.2 (1.149) |
| I am convinced that my colleagues use WhatsApp for professional purposes | Nurses* | 1  (0.7%) | 2  (1.3%) | 4  (2.7%) | 20 (13.3%) | 29  (19.3%) | 39 (26.0%) | 55 (36.7%) | 5.740 (1.287) |
|  | Physicians | 0  (0%) | 0  (0%) | 2  (1.5%) | 10  (7.6%) | 20  (15.2%) | 36 (27.3%) | 64 (48.5%) | 6.140 (1.032) |
| I am convinced that my colleagues use WhatsApp to share scientific information | Nurses* | 1  (0.7%) | 9  (6.0%) | 7  (4.7%) | 18 (12.0%) | 31  (20.7%) | 40 (26.7%) | 44 (29.3%) | 5.430 (1.490) |
|  | Physicians | 0  (0%) | 0  (0%) | 2  (1.5%) | 15 (11.4%) | 26  (19.7%) | 41 (31.1%) | 48 (36.4%) | 5.890 (1.072) |
| I am convinced that my colleagues use WhatsApp to share patient information | Nurses* | 9  (6.0%) | 23 (15.3%) | 11  (7.3%) | 22 (14.7%) | 24  (16.0%) | 32 (21.3%) | 29 (19.3%) | 4.610 (1.907) |
|  | Physicians | 4  (3.0%) | 5 (3.8%) | 6  (4.5%) | 17 (12.9%) | 28  (21.2%) | 26 (19.7%) | 46 (34.8%) | 5.440 (1.598) |
| I am convinced that my colleagues do not want to use WhatsApp for professional purposes (reverse statement) | Nurses** | 8  (5.4%) | 14 (9.4%) | 14  (9.4%) | 29 (19.5%) | 22  (14.8%) | 38 (25.5%) | 24 (16.1%) | 4.7 (1.762) |
|  | Physicians* | 9  (6.9%) | 4  (3.1%) | 13  (9.9%) | 26 (19.8%) | 17  (13.0%) | 40 (30.5%) | 22 (16.8%) | 4.88 (1.719) |

*1 missing, **2 missing

**Table S5: Risk Perception** (Statements 45-59)

| **Statement** | **Profession** | **Strongly Disagree** | **Disagree** | **Somewhat Disagree** | **Neither Agree nor Disagree** | **Somewhat Agree** | **Agree** | **Strongly Agree** | **Mean (SD)** |
| --- | --- | --- | --- | --- | --- | --- | --- | --- | --- |
| I am convinced that transmitting patient information via WhatsApp is safe and does not involve risks (reverse statement) | Nurses | 14 (9.3%) | 16 (10.6%) | 14  (9.3%) | 30 (19.9%) | 23  (15.2%) | 22 (14.6%) | 32 (21.2%) | 4.50 (1.942) |
|  | Physicians | 5  (3.8%) | 11 (8.3%) | 29  (22.0%) | 26 (19.7%) | 25  (18.9%) | 22 (16.7%) | 14 (10.6%) | 4.34 (1.615) |
| I am convinced that sending clinical data via WhatsApp involves legal risks | Nurses | 8  (5.3%) | 2  (1.3%) | 6  (4.0%) | 39 (25.8%) | 34  (22.5%) | 26 (17.1%) | 36 (23.8%) | 5.06 (1.588) |
|  | Physicians | 2  (1.5%) | 6  (4.5%) | 6  (4.5%) | 31 (23.5%) | 35  (26.5%) | 31 (23.5%) | 21 (15.9%) | 5.03 (1.398) |
| I am convinced that WhatsApp use involves risks to patients related to privacy, medical confidentiality, and data protection | Nurses | 6  (4.0%) | 3  (2.0%) | 8  (5.3%) | 33 (21.9%) | 28  (18.5%) | 31 (20.5%) | 42 (27.8%) | 5.22 (1.591) |
|  | Physicians | 5  (3.8%) | 3  (2.3%) | 12  (9.1%) | 23 (17.4%) | 41  (31.1%) | 27 (20.5%) | 21 (15.9%) | 4.95 (1.484) |
| I am convinced that WhatsApp use carries the risk of uncontrolled dissemination of sensitive data | Nurses | 3  (2.0%) | 4  (2.6%) | 6  (4.0%) | 27 (17.9%) | 35  (23.2%) | 29 (19.2%) | 47 (31.1%) | 5.40 (1.479) |
|  | Physicians | 23 (17.4%) | 5  (3.8%) | 8  (6.1%) | 28 (21.2%) | 38  (28.8%) | 27 (20.5%) | 23 (17.4%) | 5.02 (1.441) |
| I am convinced that WhatsApp use in teams is dangerous because there are no guidelines and recommendations for safe usage | Nurses | 6  (4.0%) | 6  (4.0%) | 15  (9.9%) | 33 (21.9%) | 30  (19.9%) | 30 (19.9%) | 31 (20.4%) | 4.91 (1.625) |
|  | Physicians | 6  (4.5%) | 11 (8.3%) | 16  (12.1%) | 33 (25.0%) | 31  (23.5%) | 21 (15.9%) | 14 (10.6%) | 4.45 (1.584) |
| I am convinced that WhatsApp use disrupts the team during their work and increases the risk of errors | Nurses | 15 (9.9%) | 13 (8.6%) | 23  (15.2%) | 40 (26.3%) | 26  (17.2%) | 17 (11.3%) | 17 (11.3%) | 4.11 (1.742) |
|  | Physicians | 11 (8.3%) | 39 (29.5%) | 29  (22.0%) | 29 (22.0%) | 15  (11.4%) | 4 (3.0%) | 5  (3.8%) | 3.23 (1.465) |
| I am convinced that WhatsApp use does not involve medical confidentiality risk since the information is end-to-end encrypted and therefore protected (reverse statement) | Nurses | 6  (4.0%) | 6  (4.0%) | 18  (12.1%) | 33 (22.1%) | 28  (18.8%) | 28 (18.8%) | 30 (20.1%) | 4.85 (1.643) |
|  | Physicians | 2  (1.5%) | 6  (4.5%) | 17  (12.9%) | 33 (25.0%) | 26  (19.7%) | 27 (20.5%) | 21 (15.9%) | 4.82 (1.497) |
| I am convinced that WhatsApp use does not involve medical confidentiality risk since the smartphone device is protected by password/fingerprint/face recognition (reverse statement) | Nurses | 7  (4.7%) | 7  (4.7%) | 22  (14.7%) | 29 (19.3%) | 24  (16.0%) | 25 (16.7%) | 36 (24.0%) | 4.83 (1.751) |
|  | Physicians | 3  (2.3%) | 4  (3.0%) | 18  (13.6%) | 35 (26.5%) | 22  (16.7%) | 26 (19.7%) | 24 (18.2%) | 4.84 (1.537) |
| I am convinced that WhatsApp use involves medical confidentiality risk since photos are automatically saved to the gallery and cloud | Nurses | 11 (7.3%) | 9  (6.0%) | 13  (8.6%) | 31 (20.5%) | 23  (15.2%) | 37 (24.5%) | 27 (17.9%) | 4.75 (1.781) |
|  | Physicians | 9  (6.8%) | 5  (3.8%) | 9  (6.8%) | 37 (28.0%) | 31  (23.5%) | 24 (18.2%) | 17 (12.9%) | 4.64 (1.603) |
| I am convinced that WhatsApp use constitutes a violation of patient confidentiality | Nurses | 5  (3.3%) | 14 (9.3%) | 11  (7.3%) | 36 (24.0%) | 36  (24.0%) | 29 (19.3%) | 25 (16.7%) | 4.73 (1.646) |
|  | Physicians | 6  (4.5%) | 15 (11.5%) | 20  (15.3%) | 43 (32.8%) | 22  (16.8%) | 16 (12.2%) | 9 (6.9%) | 4.10 (1.518) |
| I am concerned about the risks involved in WhatsApp use | Nurses | 12 (8.0%) | 12 (8.0%) | 13  (8.7%) | 43 (28.7%) | 34  (22.0%) | 16 (10.7%) | 21 (14.0%) | 4.37 (1.708) |
|  | Physicians | 9  (6.8%) | 15 (11.4%) | 15  (11.4%) | 44 (33.3%) | 22  (16.7%) | 21 (15.9%) | 6  (4.5%) | 4.08 (1.551) |
| I am convinced that one should strictly adhere to transmitting and receiving non-identifiable information only | Nurses | 3  (2.0%) | 5  (3.3%) | 7  (4.6%) | 23 (15.2%) | 26  (17.2%) | 22 (14.6%) | 65 (43.0%) | 5.58 (1.581) |
|  | Physicians | 2  (1.5%) | 4  (3.0%) | 8  (6.0%) | 22 (16.7%) | 29  (22.0%) | 33 (25.0%) | 34 (25.8%) | 5.33 (1.454) |
| I am convinced that one should strictly adhere to immediate deletion of identifiable information received via WhatsApp | Nurses | 1  (0.7%) | 2  (1.3%) | 8  (5.3%) | 27 (17.9%) | 25  (16.6%) | 26 (17.2%) | 62 (41.1%) | 5.64 (1.425) |
|  | Physicians | 1  (0.8%) | 7  (5.3%) | 9  (6.8%) | 27 (20.5%) | 26  (19.7%) | 36 (27.3%) | 26 (19.7%) | 5.14 (1.461) |
| I prefer using an instant messaging application that would be purchased by the hospital and have maximum security for transmitting identifiable patient material | Nurses | 6  (4.0%) | 4  (2.6%) | 12  (7.9%) | 31 (20.5%) | 17  (11.3%) | 23 (15.2%) | 28 (38.4%) | 5.32 (1.733) |
|  | Physicians | 7  (5.3%) | 15 (11.4%) | 8  (6.1%) | 32 (24.2%) | 15  (11.4%) | 27 (20.5%) | 28 (21.2%) | 4.71 (1.831) |
| If requested, I would install a secure instant messaging application purchased by the hospital on my personal smartphone | Nurses | 7  (4.6%) | 6  (4.0%) | 6  (4.4%) | 29 (19.2%) | 15  (9.9%) | 31 (20.5%) | 57 (37.7%) | 5.38 (1.743) |
|  | Physicians | 2  (1.5%) | 6  (4.5%) | 4  (3.0%) | 26 (19.7%) | 13  (9.8%) | 41 (31.1%) | 40 (30.3%) | 5.46 (1.515) |

Note: Statements 45, 51, and 52 are reverse statements
